# High-throughput sequencing data of soil bacterial communities from Tweefontein indigenous and commercial forests, South Africa

**DOI:** 10.1016/j.dib.2019.104916

**Published:** 2019-12-03

**Authors:** Adenike Eunice Amoo, Ben Jesuorsemwen Enagbonma, Olubukola Oluranti Babalola

**Affiliations:** Food Security and Safety, Faculty of Natural and Agricultural Sciences, North-West University, Private Bag X2046, Mmabatho, 2735, South Africa

**Keywords:** 16S rRNA amplicon sequencing, Anthropogenic interference, Illumina Miseq, Land-use change, Metagenomics, MG-RAST

## Abstract

In this report, the high-throughput sequencing data of soil bacterial communities from indigenous and commercial forests in Tweefontein, South Africa are presented. These data were collected to study the influence of land-use change on soil bacterial diversity and community structure in forests. Illumina Miseq sequencing of 16S rRNA gene amplicon was carried out on soils sampled from Tweefontein commercial (TC) and indigenous (TI) forests in South Africa. The metagenome contained 101,938 sequences with 46,709,377 bp size and 57% G + C content in TI and 91,160 sequences with 41,707,827 bp size and 57% G + C content in TC. Metagenome sequence information are available at NCBI under the Sequence Read Archive (SRA) database with accession numbers SRR8134476 (TI) and SRR8135323 (TC). Taxonomic hits distribution from Metagenomic Rast Server (MG-RAST) analysis of the TI sample revealed the dominance of the phyla *Acidobacteria* (21.61%), *Actinobacteria* (18.23%) and *Verrucomicrobia* (16.78%). Predominant genera were *Candidatus Koribacter* (12.82%), *Candidatus Solibacter* (11.74%) and *Chthoniobacter* (9.36%). MG-RAST assisted analysis of TC sample also detected the dominance of *Actinobacteria* (23.62%) along with *Verrucomicrobia* (21.92%) and *Acidobacteria* (20.74%). Predominant genera were *Chthoniobacter* (24.94%), *Candidatus Solibacter* (16.74%) and *Candidatus Koribacter* (9.39%) which play vital ecological functions in forest ecosystems.

**Table:** Specifications Table

Subject	Microbiology
Specific subject area	Applied Microbiology and Biotechnology
Type of data	16S rRNA amplicon sequencing data
How data were acquired	NGS sequencing on Illumina MiSeq platform
Data format	Raw data (FASTQ file)
Parameters for data collection	Environmental sample, forest soil and winter
Description of data collection	Metagenomic DNA extraction from Tweefontein forest soils, NGS sequencing on Illumina MiSeq platform and MG-RAST analysis of the NGS data
Data source location	Institution: North-West University City/Town/Region: Mafikeng, North West Province Country: South Africa Latitude and longitude (and GPS coordinates) for collected samples/data: −24°58′S, 30.48′E and 1239.47 m above mean sea level
Data accessibility	Repository name NCBI SRA Data identification number: SRR8134476 (TI) and SRR8135323 (TC) Direct URL to data: https://www.ncbi.nlm.nih.gov/sra/SRR8134476 (TI) and https://www.ncbi.nlm.nih.gov/sra/?term=SRR8135323 (TC)

**Table:** 

**Value of the data** • The data provides insight into the impact of land-use change from native forests to commercial plantations on the community structure and diversity of soil bacteria. • Bacterial communities inhabiting indigenous forest soils could serve as a reservoir of bioactive molecules and novel genes needed for industrial and biotechnological purposes. • Soil bacterial communities play important roles in the functioning of forest ecosystems as they partake in many essential processes such as carbon and nitrogen cycling. Understanding how alterations in land use affect their composition and diversity is important as it could directly affect these ecosystem functions. • In future, a larger sample size and going further to check the functional diversity of these microbial communities would reveal the implications of changes in land use for various ecosystem functions.

## 1 Data

**Fig. 1:**
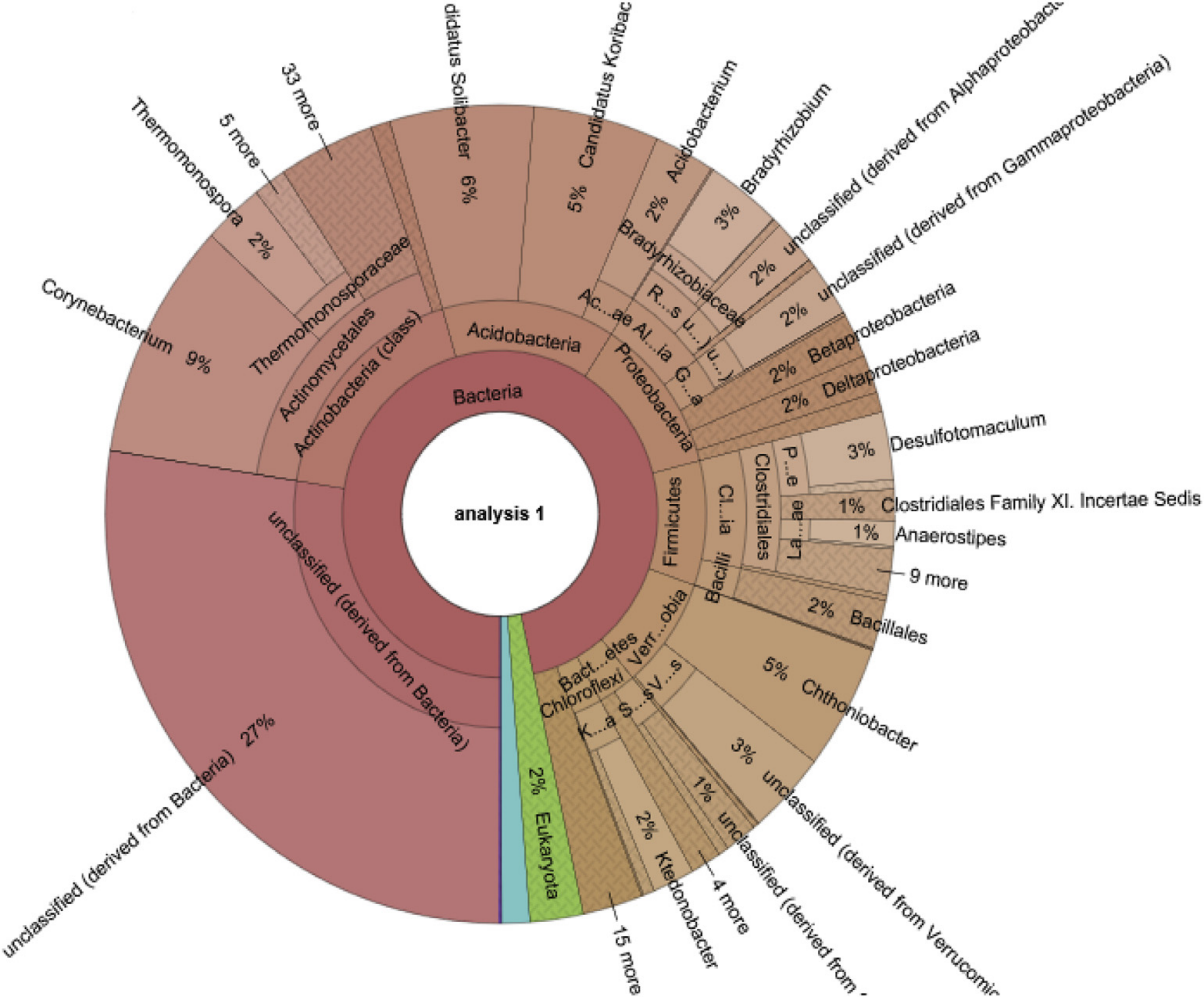
Interactive Krona chart for the visualization of bacterial communities detected from Tweefontein indigenous forest soil.

**Fig. 2:**
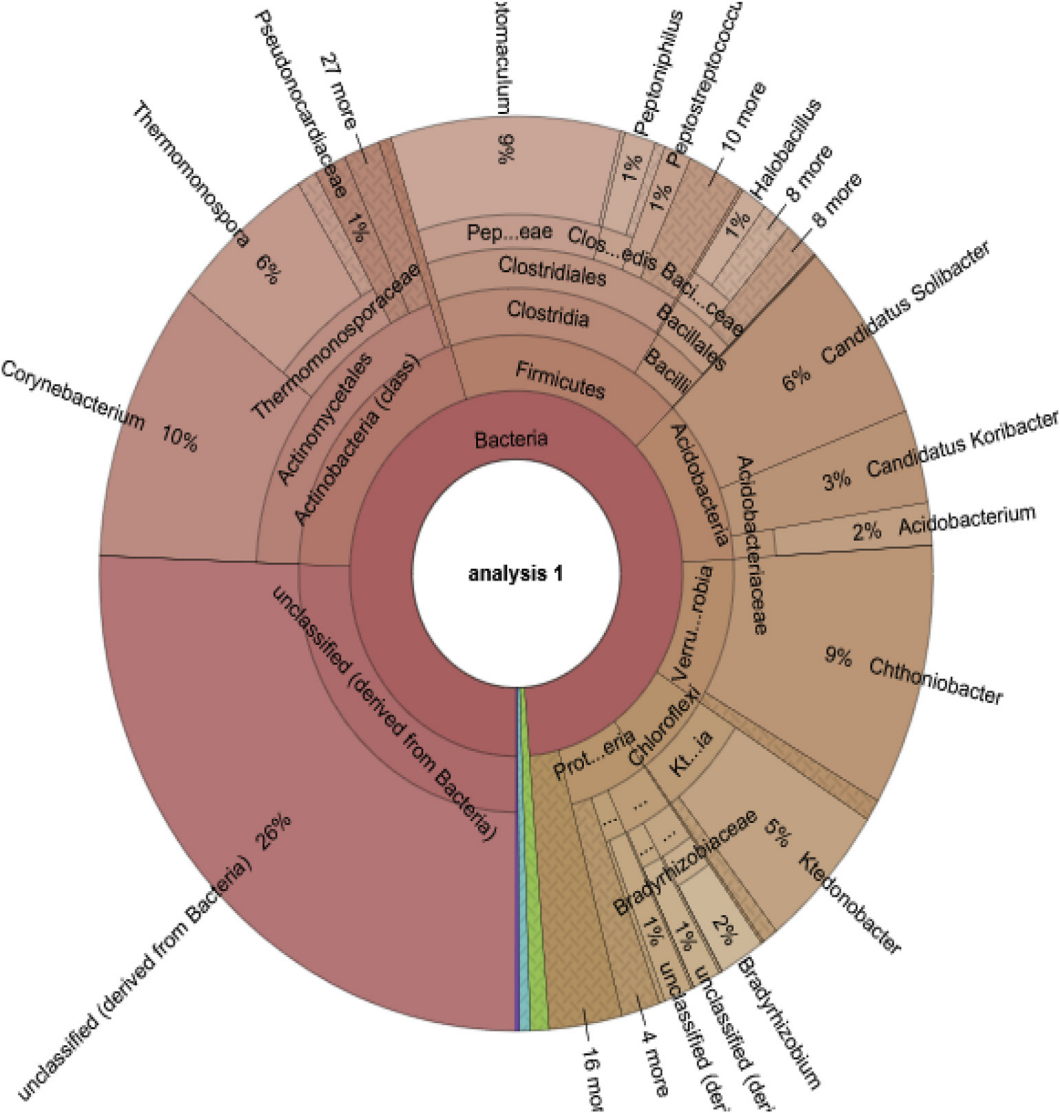
Interactive Krona chart for the visualization of bacterial communities detected from Tweefontein commercial forest soil.

The dataset contains raw sequencing data acquired through the 16S rRNA amplicon sequencing of Tweefontein indigenous (TI) and commercial forest (TC) soils from South Africa. The data files (reads in FASTQ format) were deposited at NCBI SRA database under project accession numbers SRR8134476 (TI) and SRR8135323 (TC). Data about the diversity and structure of bacterial communities of Tweefontein indigenous (TI) and commercial (TC) forest soils are presented in Fig. 1, Fig. 2 respectively.

## 2 Experimental design, materials and methods

In this dataset, soil samples were collected from Tweefontein indigenous forest and the adjacent Tweefontein commercial forest (−24°58′S, 30.48′E and 1239.47 m above mean sea level) in July 2016 during winter. The indigenous forest covers an area of 10,484.09 ha while the commercial forest covers 5965.84 ha. The commercial forest is presently on second rotation (one rotation = 30 years) and sustainable forest management is practiced. This plantation has been FSC certified for the past 20 years [1]. The indigenous forest and commercial plantation are about 2 km apart. Ten soil cores (2 cm in diameter and 10 cm in depth) were collected within multiple tree rows at various points within the sampling sites. These cores were then pooled together and homogenized into a composite sample per site. After sampling, the soil samples were preserved temporarily in cooler boxes filled with ice and conveyed to the laboratory where they were stored in a fridge at a temperature of 4°C for 2 weeks. Thereafter, metagenomic DNA extraction was performed using the PowerSoil® DNA isolation kit (MoBio Laboratory, CA, USA) according to the manufacturer's instructions. NGS was done using Illumina Miseq at Molecular Research LP, Shallowater, TX, USA. Quality and quantity of extracted DNA were analysed by NanoDrop ND-2000 and Qubit. The 16S rRNA libraries were prepared from the QC passed DNA samples using the PCR primers 515F (5′ - AATGATACGGCGACCACCGAGATCTACAC TATGGTAATT GT GTGCCAGCMGCCGCGGTAA – 3′) and 806R (5′ - CAAGCAGAAGACGGCATACGAGAT TCCCTTGTCTCC AGTCAGTCAG CC GGACTACHVGGGTWTCTAAT – 3′) with standard Illumina barcodes and adapters. The amplicons were further purified using Ampure XP beads. The barcoded libraries were validated by Agilent DNA 1000 Bioanalyser and quantified using Qubit DNA BR reagent assay. The quantified libraries were pooled and sequenced using MiSeq. Raw sequences from Illumina Miseq were processed and analysed using MG-RAST server v4.0.3 (http://metagenomics.anl.gov/) [2]. Raw data were uploaded as FASTQ files after demultiplexing of paired-end reads. Reads generated after quality processing and deduplication by MG-RAST pipeline analysis were subjected to taxonomic analysis. MG-RAST pipeline made available an estimation of bacterial abundances present in Tweefontein indigenous and commercial forests and based on this, an evaluation was done to appraise the bacterial diversity within the samples.

## 3 Nucleotide sequence accession number

Sequences used for the compilation of this data have been deposited in the Sequence Read Archive (SRA) of the National Center for Biotechnology Information (NCBI) under the bioproject numbers SRR8134476 (TI) and SRR8135323 (TC).
